# Achromatic Microscopes

**Published:** 1852-06

**Authors:** 


*Achromatic Microscopes.*

By reference to our advertising columns it will be seen that Wm. Buffham, of Millburn, Lake county, Ill., has constructed a neat, cheap working Microscope, which has all the qualities of much higher priced instruments of the same magnifying power with very superior defining power, at the low price of $15.00, and which for clearness of vision and distinctness of outline cannot, in our opinion, be surpassed. No physician, wishing to avail himself of all the modern improvements, should be without such an instrument. H.

